# Performance with robotic surgery versus 3D- and 2D­laparoscopy during pancreatic and biliary anastomoses in a biotissue model: pooled analysis of two randomized trials

**DOI:** 10.1007/s00464-021-08805-3

**Published:** 2021-11-19

**Authors:** Maurice J. W. Zwart, Leia R. Jones, Ignacio Fuente, Alberto Balduzzi, Kosei Takagi, Stephanie Novak, Luna A. Stibbe, Thijs de Rooij, Jony van Hilst, L. Bengt van Rijssen, Susan van Dieren, Aude Vanlander, Peter B. van den Boezem, Freek Daams, J. Sven D. Mieog, Bert A. Bonsing, Camiel Rosman, Sebastiaan Festen, Misha D. Luyer, Daan J. Lips, Arthur J. Moser, Olivier R. Busch, Mohammad Abu Hilal, Melissa E. Hogg, Martijn W. J. Stommel, Marc G. Besselink, for the Dutch Pancreatic Cancer Group, Luna A. Stibbe

**Affiliations:** 1 Department of Surgery, Amsterdam UMC, University of Amsterdam, Cancer Center Amsterdam, De Boelelaan 1117 (ZH-7F), 1081 HV Amsterdam, The Netherlands; 2 Department of General Surgery, Instituto Ospedaliero Fondazione Poliambulanza, Brescia, Italy; 3 Department of Surgery, Hospital Italiano de Buenos Aires, Buenos Aires, Argentina; 4 General and Pancreatic Surgery Department, Pancreas Institute, University and Hospital Trust of Verona, Verona, Italy; 5 Department of Surgery, Erasmus University Medical Center, Rotterdam, The Netherlands; 6 Department of Gastroenterological Surgery, Transplant, and Surgical Oncology, Okayama University, Okayama, Japan; 7 Department of Surgery, Hillman Cancer Center, University of Pittsburgh Medical Center, Pittsburgh, PA USA; 8 Department of Surgery, University Hospital Ghent, University of Ghent, Ghent, Belgium; 9 Department of Surgery, Radboud University Medical Center, Nijmegen, The Netherlands; 10 Department of Surgery, Amsterdam UMC, VU University, Cancer Center Amsterdam, Amsterdam, The Netherlands; 11 Department of Surgery, Leiden University Medical Center, Leiden, The Netherlands; 12 Department of Surgery, OLVG, Amsterdam, The Netherlands; 13 Department of Surgery, Catharina Hospital, Eindhoven, The Netherlands; 14 Department of Surgery, Medisch Spectrum Twente, Enschede, The Netherlands; 15 Department of Surgery, Beth Israel Deaconess Medical Center, Harvard Medical School, Boston, MA USA; 16 Department of Surgery, Northshore University Health System, Chicago, IL USA

**Keywords:** Robotic, Laparoscopy, 3D-laparoscopy, Pancreas, Liver, OSATS

## Abstract

**Background:**

Robotic surgery may improve surgical performance during minimally invasive pancreatoduodenectomy as compared to 3D- and 2D-laparoscopy but comparative studies are lacking. This study assessed the impact of robotic surgery versus 3D- and 2D-laparoscopy on surgical performance and operative time using a standardized biotissue model for pancreatico- and hepatico-jejunostomy using pooled data from two randomized controlled crossover trials (RCTs).

**Methods:**

Pooled analysis of data from two RCTs with 60 participants (36 surgeons, 24 residents) from 11 countries (December 2017–July 2019) was conducted. Each included participant completed two pancreatico- and two hepatico-jejunostomies in biotissue using 3D-robotic surgery, 3D-laparoscopy, or 2D-laparoscopy. Primary outcomes were the objective structured assessment of technical skills (OSATS: 12–60) rating, scored by observers blinded for 3D/2D and the operative time required to complete both anastomoses. Sensitivity analysis excluded participants with excess experience compared to others.

**Results:**

A total of 220 anastomoses were completed (robotic 80, 3D-laparoscopy 70, 2D­laparoscopy 70). Participants in the robotic group had less surgical experience [median 1 (0–2) versus 6 years (4–12), *p* < 0.001], as compared to the laparoscopic group. Robotic surgery resulted in higher OSATS ratings (50, 43, 39 points, *p* = .021 and *p* < .001) and shorter operative time (56.5, 65.0, 81.5 min, *p* = .055 and *p* < .001), as compared to 3D- and 2D­laparoscopy, respectively, which remained in the sensitivity analysis.

**Conclusion:**

In a pooled analysis of two RCTs in a biotissue model, robotic surgery resulted in better surgical performance scores and shorter operative time for biotissue pancreatic and biliary anastomoses, as compared to 3D- and 2D-laparoscopy.

Minimally invasive pancreatoduodenectomy (MIPD) is becoming increasingly popular [1]. Recently, the single-center Spanish PADULAP and Indian PLOT randomized controlled trials resulted in shorter hospital stay and less complications with laparoscopic as compared to open pancreatoduodenectomy [2, 3]. However, the first multicenter randomized controlled LEOPARD-2 trial was terminated early for safety concerns with laparoscopic MIPD which could have been related to a learning curve effect [4].

Robotic surgery aims to overcome the compromises made with 2D-laparoscopic surgery by improving dexterity, 3D vision, and ergonomic comfort [5–7]. Outcomes for robotic MIPD in retrospective series from expert centers seem promising, including a lower conversion rate as compared to laparoscopic MIPD [5, 8]. However, robotic surgery also has several downsides such as high costs, docking time, and loss of haptic feedback [9–12]. These shortcomings might be overcome by 3D-laparoscopy. Several authors have reported excellent outcomes with this approach [13, 14]. However, studies comparing surgical performance with robotic surgery, 3D-, and 2D­laparsocopy are currently lacking [15].

In recent years, use of artificial organs (biotissue) to improve surgical training in MIPD has gained popularity [16–20]. Since the declaration of Helsinki [21], several medical principles to safeguard the health, well-being, and rights of patients have been established, including simulation as a first step.

The aim of the present study is to assess surgical performance with robotic surgery, 3D-, and 2D­laparoscopy for the pancreatico- and hepatico-jejunostomy anastomoses of MIPD by pooling data from two previous randomized controlled crossover trials using the same standardized biotissue model.

## Materials and methods

This study was reported in accordance with the Consolidated Standards of Reporting Trials (CONSORT) [22]. Data from two previous randomized controlled crossover trials, comparing robotic surgery and 3D- and 2D-laparoscopy in a biotissue model for pancreatico- and hepatico-jejunostomy (PJ and HJ), were combined [19, 23]. The trials were registered in the Netherlands Trial Registry under code NL8063. The LAELAPS-3D2D trial compared 3D- and 2D-laparoscopy for the MIPD anastomoses [13] and the LAEBOT-3D2D trial compared robotic surgery with 3D- and 2D-vision [24]. In these trials, the participants had to complete a PJ and a HJ twice: once with 3D- and once with 2D-vision. The participants were randomized to start either with 3D- or 2D-vision in each anastomosis and had to cross-over to the other anastomosis (HJ/PJ) in other vision modality, after completing the first anastomosis. The analysis was based on individual participant data, but since the designs of the studies were highly similar, data were fully pooled. Both studies were approved by the local ethics committee and performed in accordance with the Declaration of Helsinki [21].

### Participants

Participating surgeons and residents were invited from all 17 centers collaborating within the Dutch Pancreatic Cancer Group along with related international centers. Based on sample size calculations, the LAELAPS­3D2D trial included 20 surgical experts and 20 surgical residents. None of the participating residents had performed minimally invasive pancreas procedures independently. The LAEBOT­3D2D trial included 20 participants without stratification on participant categorization as experts or residents, since no statistical difference was found in the LAELAPS­3D2D trial [13, 24]. All participants were capable of suturing with the minimally invasive approach, *i.e.,* robotic or laparoscopic*.* Participants were excluded if they had no 3D-vision abilities, as evidenced by < 200 seconds of arc on Randot Test (Stereo optical, Chicago, IL, USA) [25].

### Randomization

In both trials, randomization was done with SPSS (SPSS, Chicago, IL, USA) by the study coordinator. Participant data were anonymized using a 4-digit code and only the principal investigator and study coordinators had access to the decoding document.

### Intervention

The interventions in both trials were the same. In both trials, an identical standardized patient setting was simulated, using inanimate artificial, biotissue, organs (LifeLike BioTissue, Ontario, Canada) as previously published by King et al. [7]. The specifics on the simulation set-up and biotissue were previously published in the LAELAPS-3D2D [13] and LAEBOT-3D2D [24] trials.

For robotic surgery, the integrated 3D HD da Vinci robotic laparoscope and robotic system was used (Intuitive, Sunnyvale California, USA). For laparoscopy, the ENDOEYE FLEX 3D (Olympus, Tokyo, Japan) 10-mm articulating laparoscope with high-definition vision was used. Participants first watched an instruction video and had an oral instruction before the start of the experiment. Hereafter, participants were allocated to complete a PJ and a HJ twice in the biotissue model, once with 3D- and once with 2D-vision. The anastomosis techniques and type and number of sutures were standardized, as means to compare the groups in this pooled analysis. Resolution (high-definition/1280 × 1024) and lighting conditions were identical between both interventions and approaches.

### Blinding

Imaging material was rated by one rater who was blinded for both the performing participant and for 3D- or 2D-laparoscopy. The rater could not be blinded for robotic/laparoscopic surgery given the different instruments used. This rater was trained by SN and MH during a hands-on training in the University of Pittsburgh Medical Center. Performance was rated using an objective structured assessment of technical skills (OSATS) as validated by Birkmeyer et al. and Tam et al. [17, 26, 27]. Table 1 provides more details on the elements of the OSATS.

**Table 1:** Elements of objective structured assessment of technical skills (OSATS)

Grading definition		
1	Deficient/traumatic	
2	Lacking/lacks finesse	
3	Average	
4	Skilled	
5	Master/flawless	
Grading aspects and elucidation		
Gentleness	Gentle tissue handling that does not result in injury	
Time and motion	Fluid use of instruments without awkwardness	
Instrument handling	Economy of motion, maximum efficiency	
Flow of operation	Smooth transitions from one part of the operation to another	
Tissue exposure	Retraction that allows for good visualization and proper tissue alignment	
Summary score	Overall assessment of technical skill	

### Outcomes

Operative time was measured using video material and defined as the time between start of the first stitch to cutting the last stitch of one PJ and one HJ. Per participant, the total time to complete both PJs and HJs (i.e., time to complete four anastomoses) was taken into account. The primary outcomes were the difference in surgical performance expressed in OSATS (attainable range 12–60) and total operative time expressed in minutes and relatively in percentages. Secondary outcomes included stated side-effects and preferences as collected using questionnaires in both trials [13].

### Statistical methods

Data were analyzed using IBM SPSS statistics for Windows version 26 (IBM Corp, Armonk, NY, USA). Normally distributed continuous data are presented as means and standard deviations (SDs). Non-normally distributed continuous data are presented as medians and interquartile ranges [IQRs] or 95% confidence intervals (95%CI). Categorical (binary, nominal, and ordinal) data are presented as frequencies and percentages. Likert-Scale ordinal data are also presented in means and standard deviations, as this allows more insight into the effect size [28]. A two-tailed *p* value < 0.05 was considered statistically significant. Missing data were corrected by excluding the corresponding missing part of the video of both the intervention and control procedure into the analysis.

Baseline demographics were compared with Student’s t-test for normally distributed data, Chi-squared test for frequencies in one or more categories, and Mann–Whitney U test for non-normally distributed data. The primary outcomes were analyzed according to Mann–Whitney U test because the samples were independent. To assess the impact of surgical experience, a sensitivity analysis excluded participants with > 7 years experience (upper limit of experience in the group with the lowest experience) and experience in MIPD. Lastly, a sensitivity analysis was performed excluding those who participated in both trials.

## Results

### Participants

**Fig. 1:**
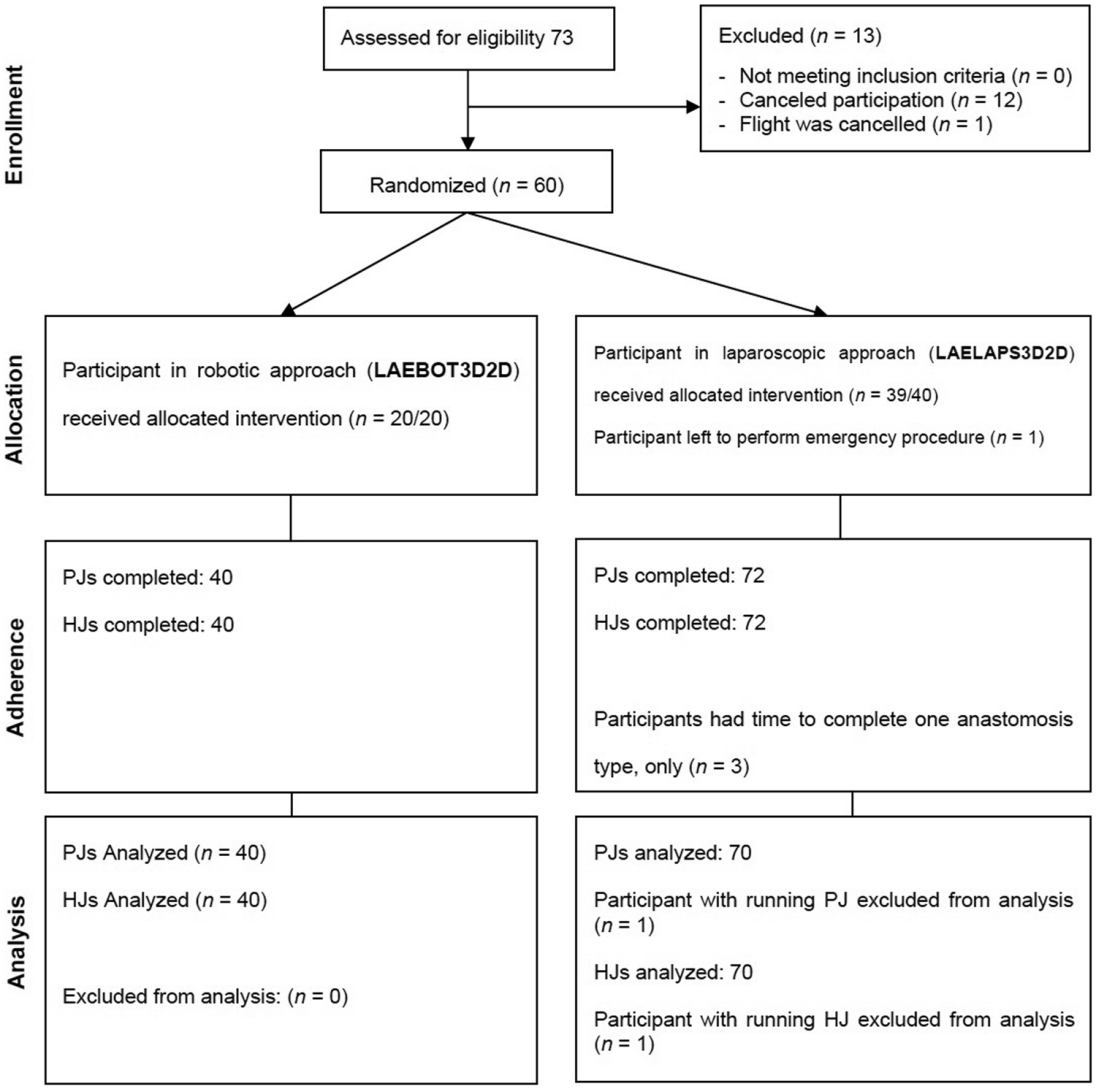
Flowchart of Inclusion for Primary Outcomes

A total of 34 surgeons and 26 residents participated and subsequently performed anastomoses between December 2017 and June 2019. Three participants only completed one anastomosis, one participant left to perform an emergency procedure, and one participant performed the anastomoses in a running fashion. These five participants were excluded for the analysis of the primary and secondary outcome. Eight participants participated in both the robotic and laparoscopic trials. Figure 1 provides an overview of the inclusion and exclusion of participants in a flow chart.

### Baseline demographics

The 55 included participants originated from 11 countries (Argentina, Belgium, Estonia, Israel, Italy, Japan, the Netherlands, South Africa, Spain, UK, and USA). Their mean age was 38 years (SD 9), 45 were male (80.0%). The groups for robotic surgery and laparoscopy were comparable for age, sex, hand dominance, the number of MIPD performed in clinical practice, and stereopsis abilities. Participants in the robotic surgery group had less experience with robotic surgery (median 1 [0–2] versus 6 years [4–12], *p* < 0.001), including a lower number of annual advanced minimally invasive procedures (median 20 versus 40, *p* = 0.014), compared to the experience of the laparoscopic group with laparoscopic surgery. In total, 15 participants from the laparoscopic group had more than 7 years experience vs none in the robotic group. Table 2 provides an overview of baseline demographics of the participants and their subgroups of robotic surgery and laparoscopic surgery.

**Table 2:** Participant characteristics

	Total (*n* = 55)	Robotic surgery (*n* = 20)	Laparoscopic surgery (*n* = 35)	*p* value
Age, mean, SD	38 ± 9	36 ± 7	39 ± 9	0.146^c^
Male, n (%)	45 (81.8)	16 (80.0)	29 (82.9)	0.606^b^
Surgical experience				
Years of experience with approach, median [IQR]	4 [1–7]	1 [0–2]	6 [4–12]	< 0.001^c^
Expert^e^ (*n* = 30)		1 [1–2]	13 [9–16]	< 0.001^c^
Resident^r^ (*n* = 25)		0 [0–1]	4 [3–5]	0.005
Annual volume of advanced MI procedures*, median [IQR]	20 [0–50]	20 [1–40]	40 [10–90]	0.014^c^
MIPDs performed, median [IQR]	0 [0–10]	0 [0–20]	0 [0–3]	0.144^c^
Hand dominance, n (%)				0.777^b^
Right	44 (80.0)	17 (85.0)	27 (77.1)	
Left	7 (12.7)	2 (10.0)	5 (14.3)	
Ambidextrous	4 (7.3)	1 (5.0)	3 (8.6)	
Vision correction, n (%)	24 (43.6)	9 (45.0)	15 (42.9)	0.877^b^
Minimal degrees of stereopsis	60 [20–100]	60 [20–100]	60 [40–100]	0.924^c^

Values are mean ± SD, median [quartile 1–quartile 3] or *n* (percentage)

^a^Student’s t­test, ^b^Chi­square test, ^c^Mann–Whitney U Test, *Minimally invasive surgery beyond appendectomy and cholecystectomy, ^e^experience as primary surgeon, ^r^experience assisting or under direct supervision of primary surgeon

### Primary outcomes

**Fig. 2:**
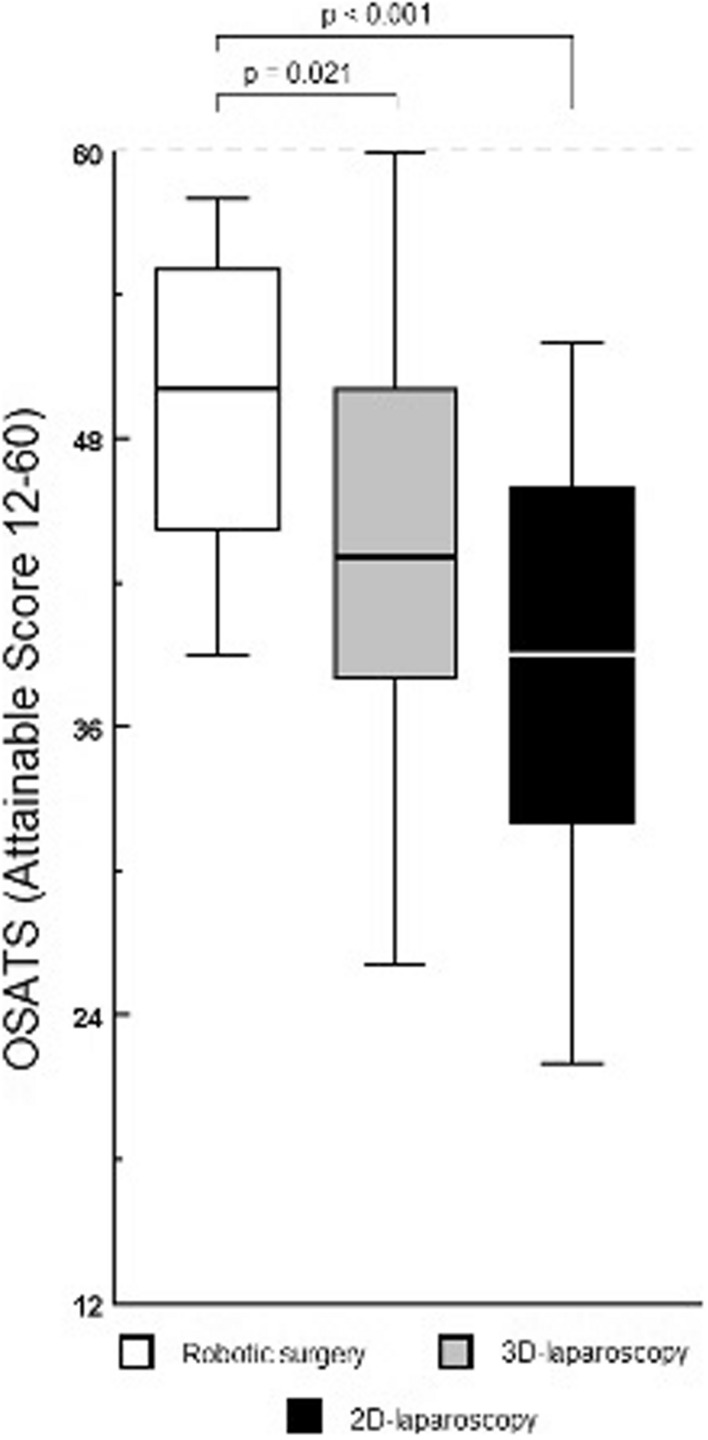
Objective Structured Assessment of Technical Skills (OSATS) for robotic surgery, 3D-, and 2D-laparoscopy to complete biotissue pancreatico- and hepatico-jejunostomy anastomoses. Legend: From left to right: first, 3D-robotic surgery (*n* = 20); second, 3D-laparoscopy (*n* = 35); third, 2D-laparoscopy (*n* = 35)

In the robotic surgery group, higher OSATS ratings were obtained (attainable range 12–60) as compared to 3D- and 2D­laparoscopy, median 50 [44–55] vs 43 [38–50] vs 39 [32–46]. Robotic surgery resulted in higher OSATS ratings by 7 points (18.4%, *p* = 0.021) and 11 points (28.2%, *p* < 0.001), as compared to 3D- and 2D­laparoscopy, respectively. Figure 2 provides an overview of the OSATS scores for robotic surgery, and 3D- and 2D-laparoscopy.

**Fig. 3:**
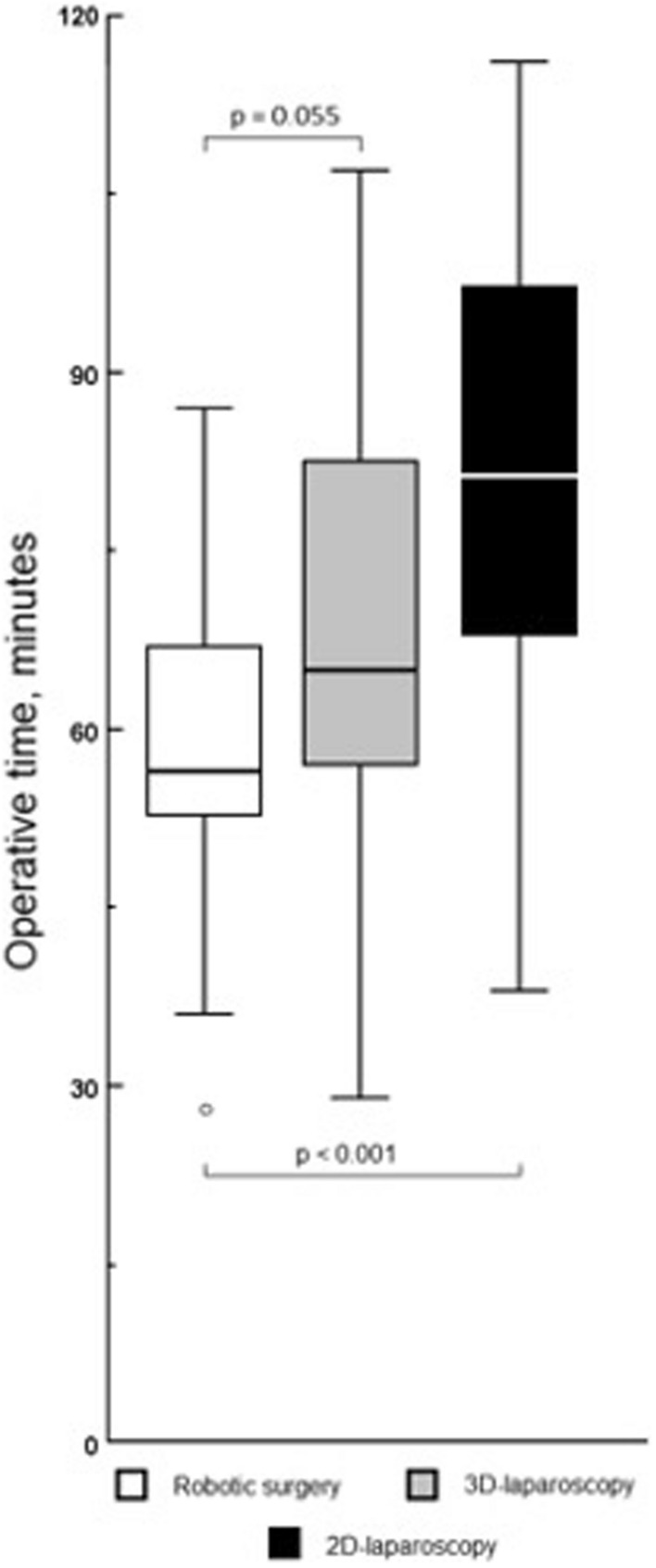
Operative time with robotic surgery, 3D-, and 2D-laparoscopy to complete biotissue pancreatico- and hepatico-jejunostomy anastomoses. Legend: From left to right: first, 3D-robotic surgery (*n* = 20); second, 3D-laparoscopy (*n* = 35); third, 2D-laparoscopy (*n* = 35)

In the robotic surgery group, operative time was shorter as compared to 3D- and 2D-laparoscopy: 56.5 min [52.4–67.5] vs 65.0 min [57.0–83.0] vs 81.5 min [68–97.8], *p* < 0.001. In the robotic surgery group, operative time was shorter by 13.1% (*p* = 0.055) and 30.7% (*p* < 0.001), as compared to 3D- and 2D-laparoscopy. Figure 3 provides an overview of the operative times for robotic surgery as compared to laparoscopy, highlighting the superiority in robotic surgery.

### Secondary outcomes

Of the 59/60 participants (20 robotic, 39/40 laparoscopic) who completed the survey on side-effects and preferences, 57/59 (96.6%) preferred 3D-vision over 2D-vision (20/20 robotic, 37/39 laparoscopic). In the 3D-robotic group, 4/20 (20%) participants reported one or more side-effects, *i.e.*, eye strain (minor *n* = 2), headache (minor *n* = 1, serious *n* = 1), dizziness (minor *n* = 2, serious *n* = 1). In the 3D-laparoscopy group, 14/39 (36%) participants reported one or more side-effects. No significant differences were found between the two groups. Table 3 provides an overview on the complaints caused by 3D vision, highlighting the severity and the robotic and laparoscopic subgroups.

**Table 3:** Complaints caused by 3D vision

	None	Minor	Moderate	Serious	Severe
Eye strain					
Laparoscopic, *n* (%)	29 (74.4)	8 (20.5)	0 (0)	0 (0)	2 (5.1)
Robotic, *n* (%)	18 (90.0)	2 (10.0)	0 (0)	0 (0)	0 (0)
Headache					
Laparoscopic, *n* (%)	36 (92.3)	2 (5.1)	0 (0)	1 (2.6)	0 (0)
Robotic, *n* (%)	18 (90.0)	1 (5.0)	0 (0)	1 (5.0)	0 (0)
Dizziness					
Laparoscopic, *n* (%)	33 (84.6)	4 (10.3)	2 (5.1)	0 (0)	0 (0)
Robotic, *n* (%)	17 (75.0)	2 (10.0)	0 (0)	1 (5.0)	0 (0)

	Yes	No
Disorientation		
Laparoscopic, *n* (%)	4 (10.3)	35 (89.7)
Robotic, *n* (%)	0 (0)	20 (100)
Physical discomfort		
Laparoscopic, *n* (%)	1 (2.6)	38 (97.4)
Robotic, *n* (%)	1 (5.0)	19 (95.0)
Poor visualization		
Laparoscopic, *n* (%)	0 (0)	39 (100)
Robotic, *n* (%)	0 (0)	20 (100)
Preferred 3D		
Laparoscopic, *n* (%)	37 (94.9)	2 (5.1)
Robotic, *n* (%)	20 (100)	0 (0)

Completed survey, lap *n* = 39, robot *n* = 20

Adapted from Zwart et al. 2019 [13], and Zwart et al. 2020 [24]

For the PJ, robotic surgery resulted in better OSATS ratings compared to 3D- and 2D-laparoscopy: 24 points [20–27] vs 22 points [18–25] vs 19 points [15–24]. Relatively, in the robotic surgery group, OSATS ratings were higher by 11.1% (*p* = 0.004) and 27.8% (*p* = 0.174), as compared to 3D- and 2D­laparoscopy. Robotic surgery resulted in shorter operative time compared to 3D- and 2D-laparoscopy: 37.5 min [30.5–43.8] vs 39.5 min [35.5–50.8] vs 50.0 min [38.0–59.0]. Relatively, in the robotic surgery group, operative time was shorter by 5.1% (*p* = 0.176) and 22.0% (*p* = 0.001), as compared to 3D- and 2D­laparoscopy.

For the HJ, robotic surgery resulted in better OSATS ratings compared to 3D- and 2D-laparoscopy: 27 points [26–29] vs 21 points [18–27] vs 18 points [16–24]. Relatively, in the robotic surgery group, OSATS ratings were higher by 28.6% (*p* < 0.001) and 42.9% (*p* = 0.002), as compared to 3D- and 2D­laparoscopy. Robotic surgery resulted in shorter operative time compared to 3D- and 2D-laparoscopy: 19.5 min [16.4–27.2] vs 25.0 min [19.0–34.0] vs 32.0 min [23.0–44.0]. Relatively, in the robotic surgery group, operative time was shorter by 22.0% (*p* = 0.061) and 39.1% (*p* < 0.001), as compared to 3D- and 2D-laparoscopy.

### Sensitivity analysis

The sensitivity analysis excluded 15 participants with experience > 7 years (upper limit of experience in the group with the lowest experience, the robotic group), all in the laparoscopy group. Table 4 provides the details on the participants’ characteristics of the sensitivity analysis, highlighting the differences between subgroups. Baseline characteristics were comparable, yet years of experience with the approach remained significantly higher in the laparoscopic group (*p* < 0.001). However, number of MIPDs performed was significantly higher (*p* = 0.023) in the robotic group, even though the median number was 0 in both groups.

**Table 4:** Sensitivity analysis excluding participants with > 7 years experience

	Total (*n* = 40)	Robotic surgery (*n* = 20)	Laparoscopic surgery (*n* = 20)	*p* value
Age, mean, SD	36 ± 7	38 ± 9	40 ± 8	0.343^a^
Male, n (%)	31 (77.5)	16 (80.0)	15 (75.0)	0.70^b^
Surgical experience				
Years of experience with approach, median [IQR]	2 [1–5]	1 [0–2]	5 [3–6]	< 0.001^c^
Expert^e^		1 [1–2]	6 [6-NA]	< 0.001^c^
Resident^r^		0 [0–1]	4 [3–6]	0.013
Annual volume of advanced MI procedures*, median (IQR)	15 [1–40]	20 [1–40]	15 [3–48]	0.752^c^
MIPDs performed, median (IQR)	0 [0–1]	0 [0–20]	0 [0–0]	0.023^c^
Hand dominance, n (%)				-^b^
Right	34 (85.0)	17 (85.0)	17 (85.0)	
Left	4 (10.0)	2 (10.0)	2 (10.0)	
Ambidextrous	2 (5.0)	1 (5.0)	1 (5.0)	
Vision correction, n (%)	17 (42.5)	9 (45.0)	8 (40.0)	0.10^b^
Minimal degrees of stereopsis	60 [20–100]	60 [20–100]	60 [40–100]	0.812^c^

Values are mean ± SD, median [quartile 1–quartile 3] or *n* (percentage)

^a^Student’s t­test, ^b^Chi­square test, ^c^Mann-Whitney U Test, *Minimally invasive surgery beyond appendectomy and cholecystectomy, ^e^experience as primary surgeon, ^r^experience assisting or under direct supervision of primary surgeon

In the sensitivity analysis, the robotic approach still had a superior OSATS as compared to 3D- and 2D-laparoscopy, median 50 [44–55] vs median 35 [27–45] vs median 31 [26–36]. Robotic surgery resulted in higher OSATS ratings by 15 points (52.1%, *p* = 0.009) and 19 points (60.4%, *p* = 0.006). The robotic approach still significantly resulted in shorter operative times: 56.5 min [52.4–67.5] vs 69.0 min [57.8–82.3] vs 81.0 min [65.0–95.5]. In the robotic surgery group, operative time was shorter by 12.4% (*p* = 0.003) and 30.2% (*p* < 0.001).

In an additional sensitivity analysis, we also excluded participants with experience in MIPD. After excluding participants with more than 7 years experience and experience in MIPD, the primary outcomes remained significantly better in the robotic group vs the 3D-laparoscopy group (OSATS ratings *p* = 0.005 and operative time *p* = 0.008). Finally, we excluded participants who performed in both the LAELAPS-3D2D and LAEBOT-3D2D trial (n = 8). The results for the primary and secondary outcome remained consistent.

## Discussion

This pooled analysis of two randomized controlled crossover trials found that robotic 3D-surgery resulted in better OSATS ratings and shorter operative time as compared to both 3D- and 2D­laparoscopy in completing PJ and HJ anastomoses in a biotissue model. Although 3D-laparoscopy improved surgical performance for both operative time and OSATS as compared to 2D-laparoscopy, robotic surgery provided additional benefits. Furthermore, fewer additional side-effects of 3D-vision were found in the robotic group compared to the 3D-laparoscopy group.

A 2017 worldwide survey on opinions and use of MIPD found that 35% of the participants felt that robotic surgery was superior to (2D) laparoscopic surgery and 64% of the participants stated that the reason for superiority was, among other reasons, 3D-vision [29]. Since this study also included 3D-laparoscopy, the shorter operative time and better OSATS ratings provided by robotic surgery were due to other elements of the robot: the wristed articulating instruments with stability and scaling control, the 3rd and 4th arm (scope) of the robot, the surgeon’s control of the camera, elimination of tremor, and ergonomic console. Robotic pancreatoduodenectomy was compared to laparoscopy in several clinical studies [15, 30]. In these, the effect of 3D-vision was largely ignored or overlooked. The majority of these studies found faster operative times in the robotic group, accompanied by similar postoperative complications [15].

By looking at expert and fellow performance using the same biotissue model, Tam et al. determined that the biotissue operative time can be generalized to clinical surgical performance [16]. Similarly, biotissue OSATS scores also translated to clinical surgical performance [27, 31] and were predictive for postoperative outcomes such as complications [27, 32, 33]. These studies used a PJ according to the modified Blumgart approach, since literature on the OSATS is validated for that method only [31]. It is unclear whether these results could be extrapolated to other types of anastomoses. Literature on colorectal and bariatric surgery suggest that minimally invasive surgical experience in years and volume impacts both operative and clinical outcomes [27, 32]. The experience in the present study was a median of 4 years, with a median of 20 advanced minimally invasive procedures annually, while experience up to 10 years and increasing procedure volume is reported to improve outcomes [34–37]. It is clear that the present study is reporting in the learning curve phase for many surgeons, especially robotic surgeons, and should ideally be repeated at a later point*, i.e.,* with further implementation [38, 39].

Robotic instrumentation provided a major contribution to surgical performance in this experimental setting. However, as reviewed by Anderson et al. elements of the robotic platform can be applied to laparoscopic surgery as well, this may be relevant in order to reduce the high cost associated with robotic surgery [40]. One study from the USA, however, reported similar costs for robotic MIPD and open pancreatoduodenectomy when taking also the costs of complications and follow-up into account [41].

The results of this study should be interpreted in light of several limitations. First, the randomized trials were performed in an experimental setting using a biotissue model and not in clinical practice. In clinical practice, clearly much more situational variation would have been observed. However, the previously mentioned studies [16, 42] clearly demonstrate the clinical value of operative time and OSATS rating using a highly standardized biotissue model. Furthermore, the biotissue anastomoses were similar to the UPMC biotissue training program for robot pancreatoduodenectomy [16, 17]. The UPMC group demonstrated that the implementation of their biotissue training program resulted in continued improvements of operative performance and patient outcomes after integration of trainees and expansion of selection criteria [34]. Several other studies have also suggested that the outcomes from biotissue simulation in pancreatoduodenectomy can support clinical performance [17–20, 23, 43]. Second, a large difference in experience was seen between participants in the robotic surgery and laparoscopic surgery groups. Even with less experience, still better outcomes were seen in the robotic surgery group. The sensitivity analysis revealed that the primary outcomes remained consistent despite this heterogeneity. Future studies including participants with more experience should determine the ‘optimal’ outcomes and the exact impact of the learning curve effect. Third, the robotic group and laparoscopic group performed the biotissue anastomoses in the same time period. However, some of the participants of the robotic group completed virtual reality simulation exercises, and this could have had an influence on the outcomes. Additionally, eight participants participated in both the laparoscopic and robotic trials which could have introduced bias due to familiarity with the setup and handling of the biotissue. Therefore, we excluded these participants in a sub-analysis and the results for the primary and secondary outcome remained consistent. Fourth, this study is not a paired comparison assessing one surgeon’s platform in relation to another but a pooled analysis, so there could be some selection bias involved but this is a better “real world” comparison of a platform. Fifth, several other differences between robotic and laparoscopic surgery could not be controlled for, for instance, better ergonomics for the surgeon in the robotic approach. We cannot exclude the possibility that better ergonomics was (partly) responsible for the better performance with the robotic approach.

Strengths of this study include the pooling of data from two randomized controlled crossover trials, thus controlling for known and unknown confounders.

In conclusion, the present study demonstrated that robotic surgery provides additional benefits over 3D- and 2D-laparoscopy when creating pancreatic and biliary anastomoses in a biotissue model. Future randomized studies should confirm these benefits in the clinical setting.
